# Supplementary material for: Manufacture-friendly nanostructured metals stabilized by dual-phase honeycomb shell
Source: Nat Commun. 2022 Apr 19;13:2034. doi: 10.1038/s41467-022-29782-8 (PMC9019002; doi:10.1038/s41467-022-29782-8)
Supplement: Supplementary file 1 — Supplementary Information [file 41467_2022_29782_MOESM1_ESM.pdf]

Supplementary Information for Manufacture-friendly nanostructured metals stabilized by dual-phase honeycomb shell

Hai Wang[1], Wei Song[1,2], Mingfeng Liu[2,3], Shuyuan Zhang[1], Ling Ren[1]*, Dong Qiu[4]*, Xing-Qiu Chen[3], Ke Yang[1]

[1]Shi-changxu Innovation Center for Advanced Materials, Institute of Metal Research, Chinese Academy of Sciences, Shenyang, China.

[2]School of Materials Science and Engineering, University of Science and Technology of China, Shenyang, China

[3]Shenyang National Laboratory for Materials Science, Institute of Metal Research, Chinese Academy of Sciences, Shenyang, China.

[4]Centre for Additive Manufacturing, School of Engineering, RMIT University, Melbourne, Victoria, Australia.

*Correspondence to: Ling Ren, lren@imr.ac.cn. Dong Qiu, dong.qiu2@rmit.edu.au

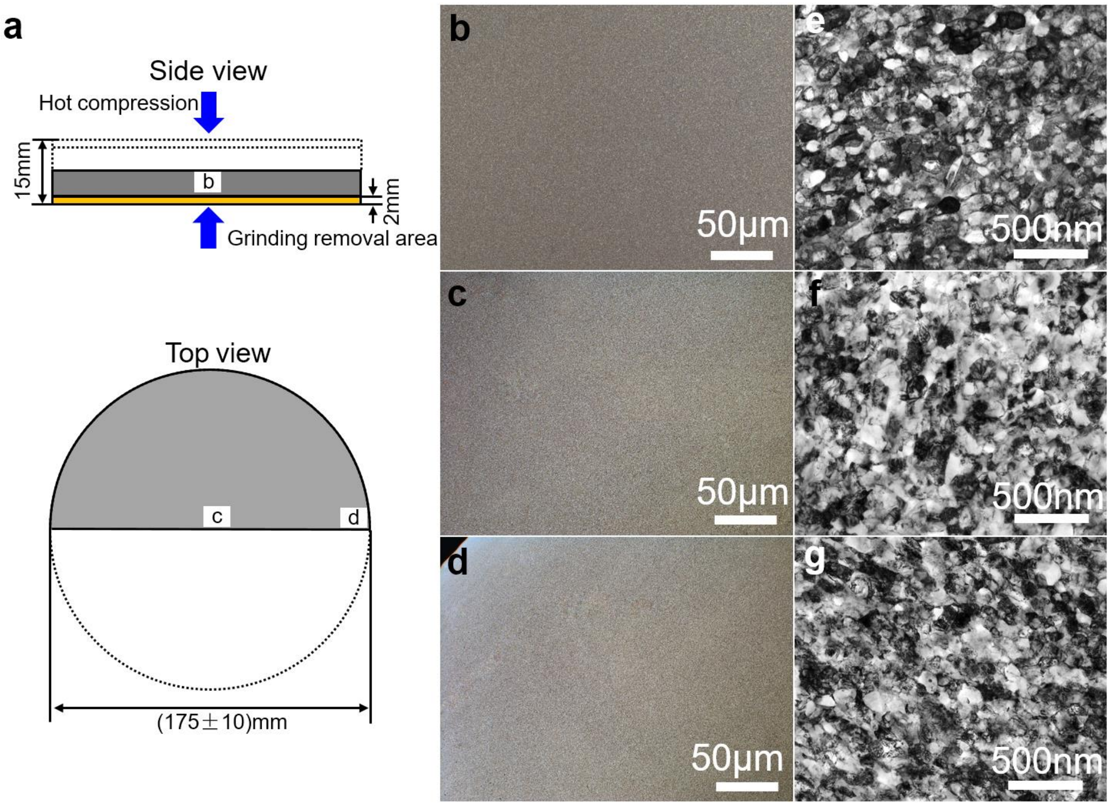

Supplementary Fig. 1 As-fabricated nanostructured Ti6Al4V5Cu alloy. **a,** Sample profile overview. **b, c** and **d,** Optical micrographs at selected areas. **e, f** and **g,** TEM images of the microstructure in areas **b, c** and **d,** respectively.

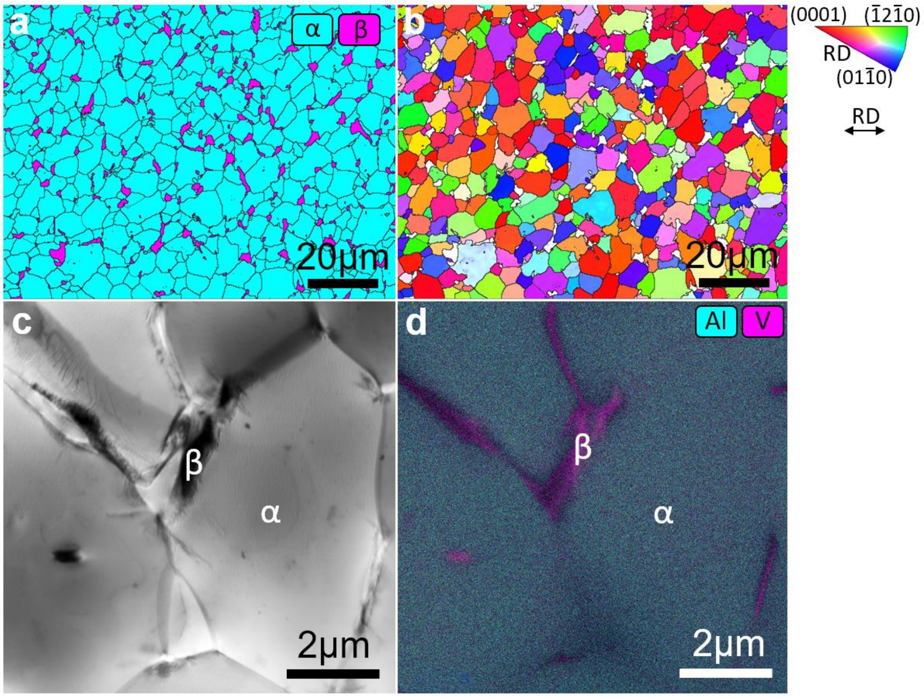

Supplementary Fig. 2 The microstructure of commercial Ti6Al4V base alloy consisting of equiaxed α grains. **a,** EBSD phase distribution map and **b**, α phase IPF map. Rolling direction (RD) is denoted in the figure. **c,** Bright field TEM image close to an α grain boundary and **d**, TEM-EDS map of the area of **c**.

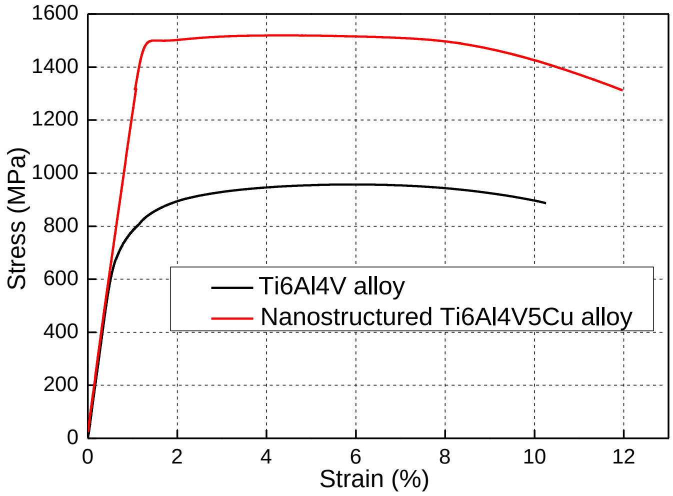

Supplementary Fig. 3 Tensile curves of the as-prepared Ti6Al4V5Cu and Ti6Al4V alloy.

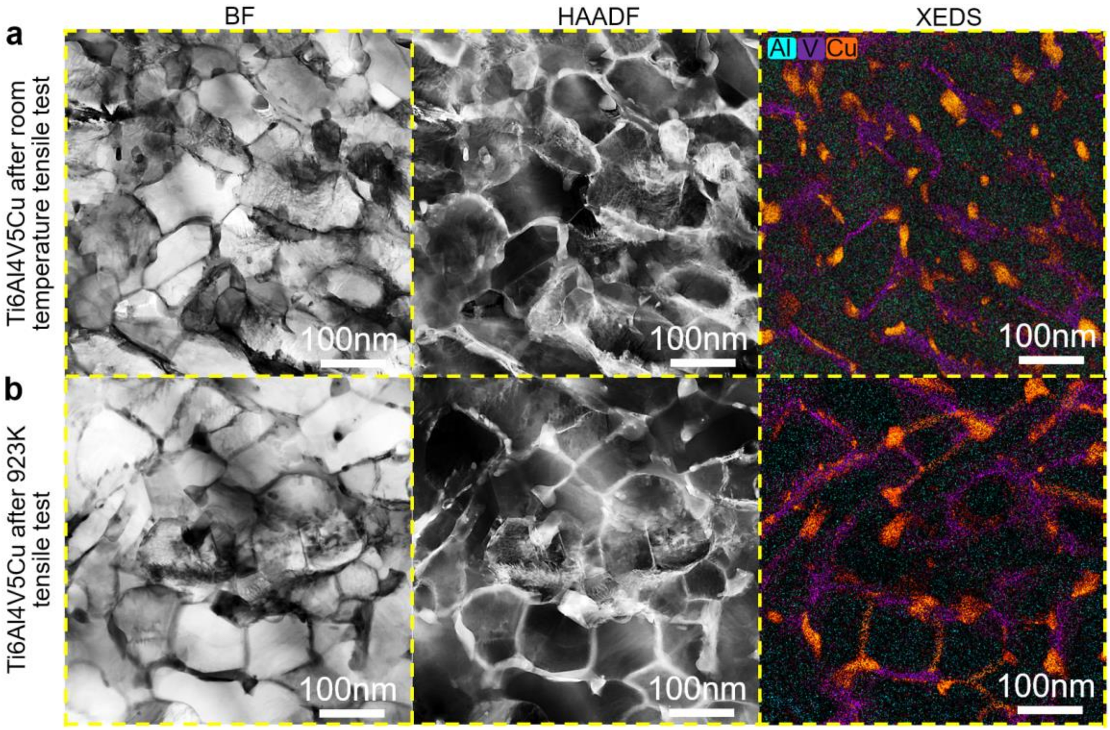

Supplementary Fig. 4 TEM observation of the nanostructured Ti6Al4V5Cu alloy. **a,** After room temperature tensile test. **b,** After 923 K tensile test with a deformation strain of exceeding 1000%. Images were taken under bright field (BF), high angle annular dark field (HAADF), and X-ray energy dispersive spectroscopy (XEDS) mode. The nanostructure was barely evolved after room and high temperature tensile tests, indicating the material possessed excellent mechanical and thermomechanical coupling stability.

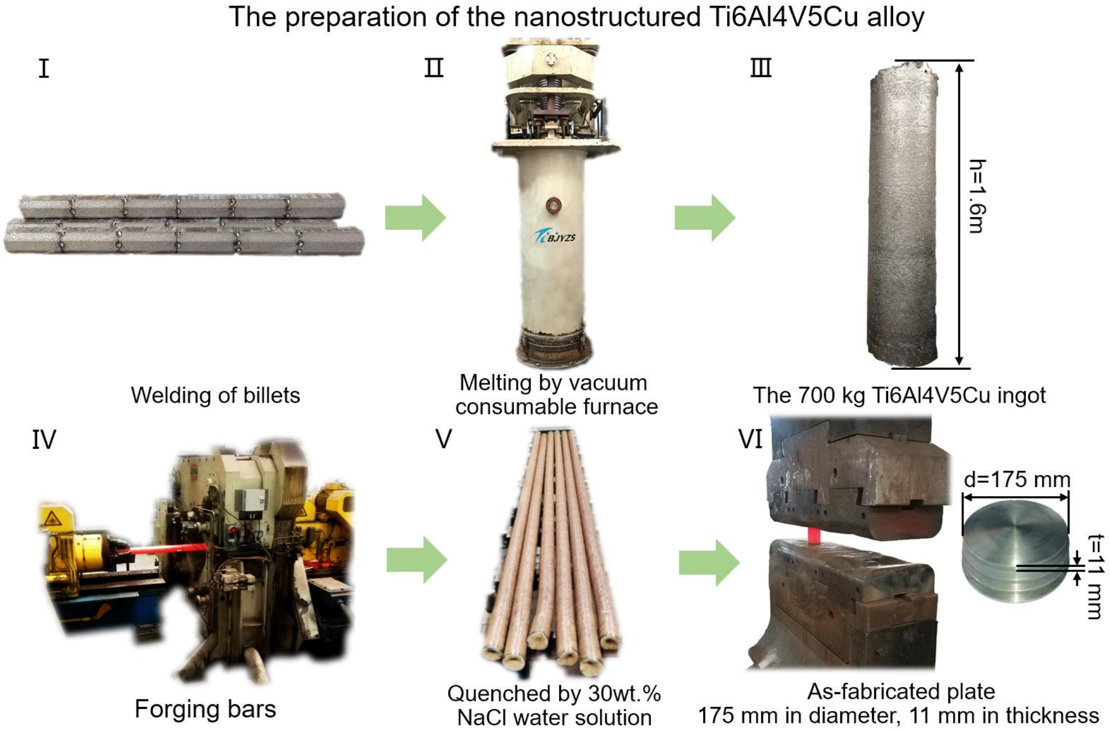

Supplementary Fig. 5 Fabrication procedures for the nanostructured Ti6Al4V5Cu alloy

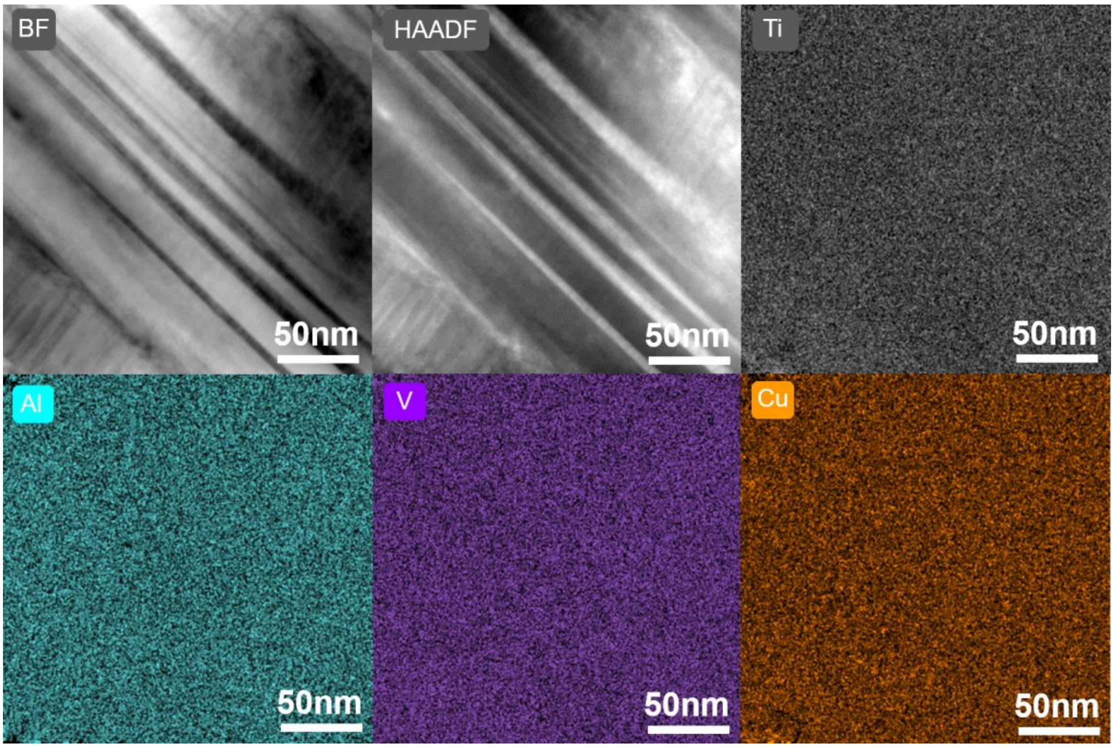

Supplementary Fig. 6 TEM observation of the as-quenched Ti6Al4V5Cu alloy. XEDS maps show that elements are homogenously distributed in the material.

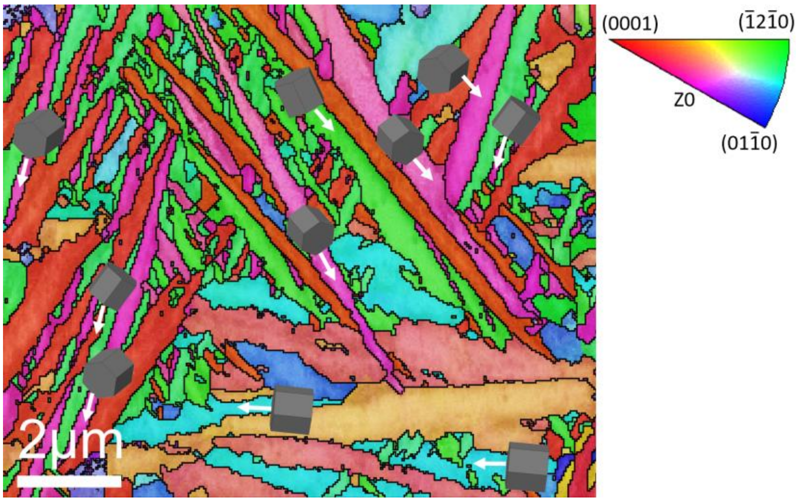

Supplementary Fig. 7 IPF maps of as-quenched Ti6Al4V5Cu alloy. All the prismatic planes of hcp α phase are parallel with the longitudinal direction of laths.

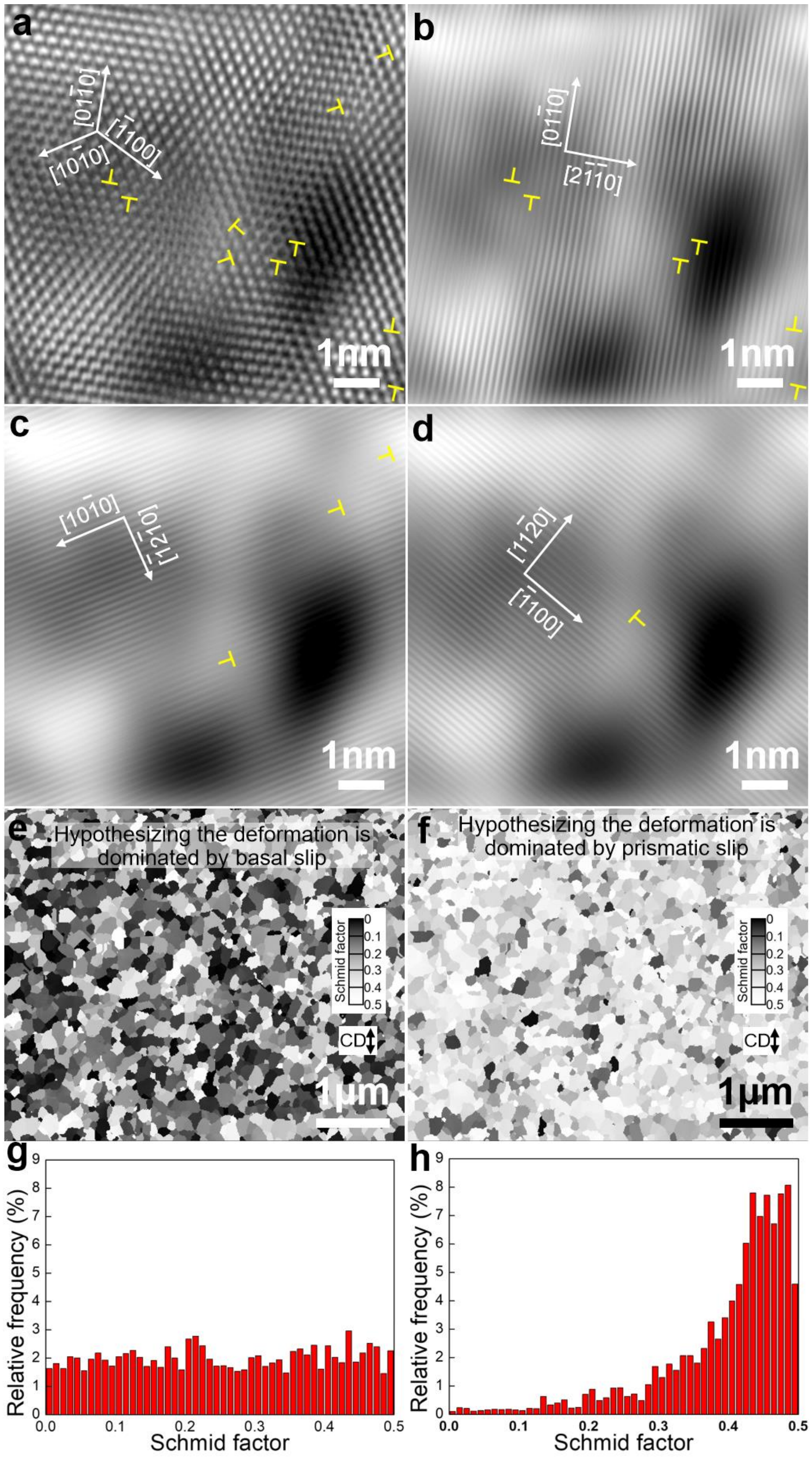

Supplementary Fig. 8 Identification of the dominant deformation mode during the formation of DPHS nanostructured Ti6Al4V5Cu alloy. **a,** High resolution TEM image in a local α grain with the zone axis of <0002>. **b**, **c**, and **d**, Simulated lattice fringes of each prismatic plane in {1 1 $\bar{2}$ 0} family to show the edge component of <a> type dislocations lying in {1 $\bar{1}$ 0 0} prismatic planes. **e** and **f,** EBSD Schmid factor maps hypothesizing the hot deformation is dominated by basal slip and prismatic slip, respectively. **g** and **h**, Frequency histogram of Schmid factors in **e** and **f** respectively.

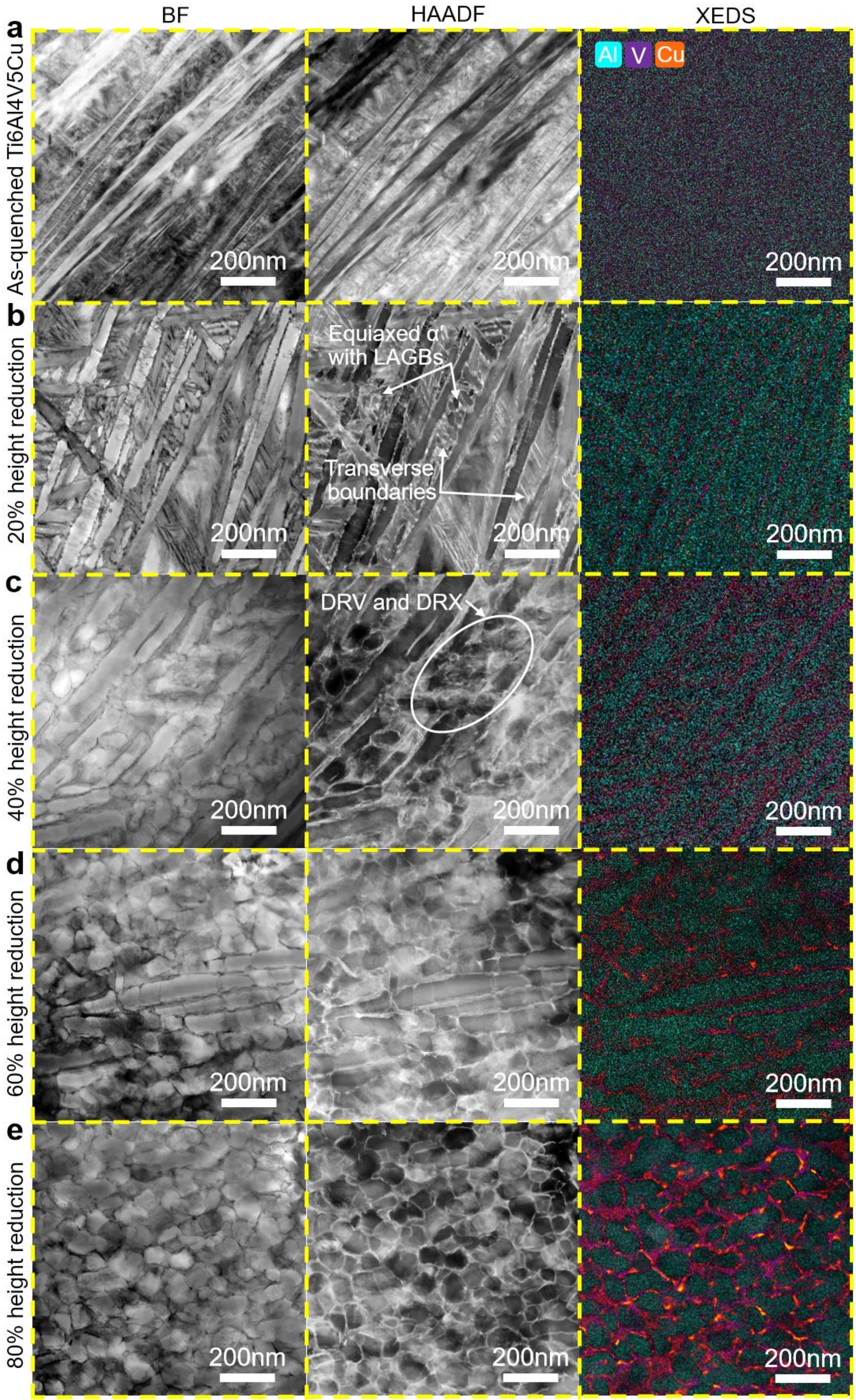

Supplementary Fig. 9 TEM/EDS analysis of the microstructure at different stages during hot compression. **a,** Before hot compression. **b,** At 20% compression strain, transverse boundaries were introduced through prismatic slip, and equiaxed α′ grains with low angle grain boundaries (LAGBs) formed. **c,** At 40% compression strain, dynamic recovery (DRV) and dynamic recrystallization (DRX) took place, and LAGBs evolved into high angle grain boundaries (HAGBs). **d, e,** At 60%~80% compression strain, conjugated β and Ti_2Cu phases massively precipitated along the HAGBs, forming the DPHS nanostructure.

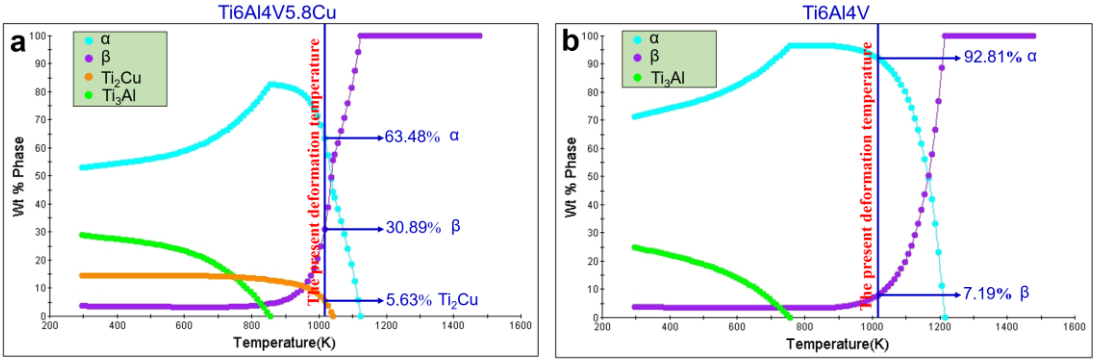

Supplementary Fig. 10 Equilibrium phase diagram calculated by JMatPro software. **a,** Ti6Al4V5Cu alloy. **b,** Ti6Al4V alloy.

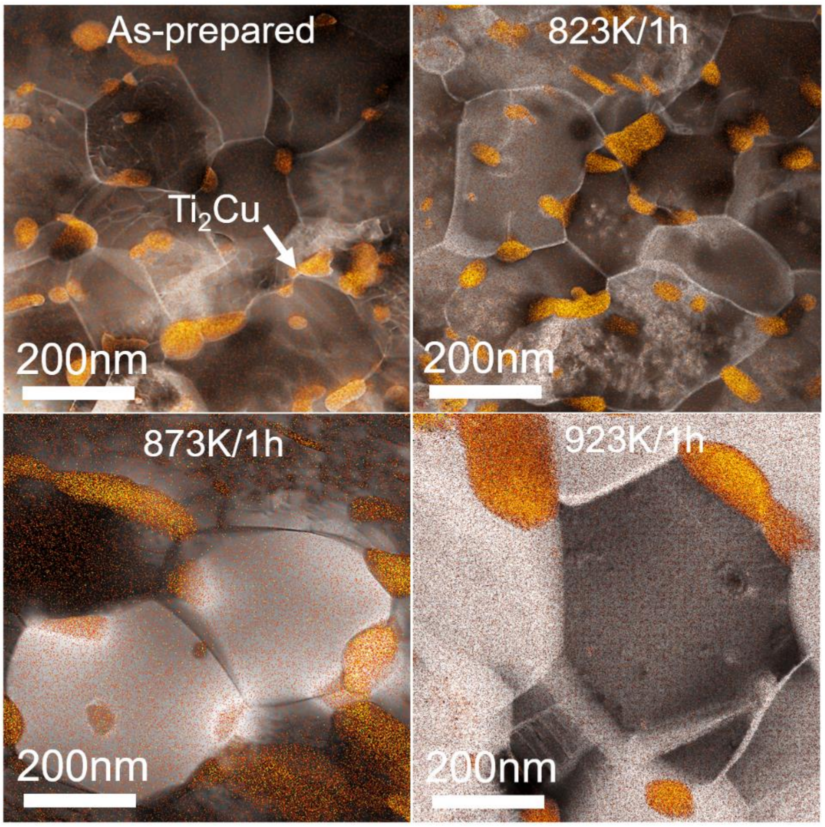

Supplementary Fig. 11 Thermal stability against annealing of the nanostructured Ti5Cu alloy. It showed inferior stability against annealing than the Ti6Al4V5Cu alloy with DPHS nanostructure.

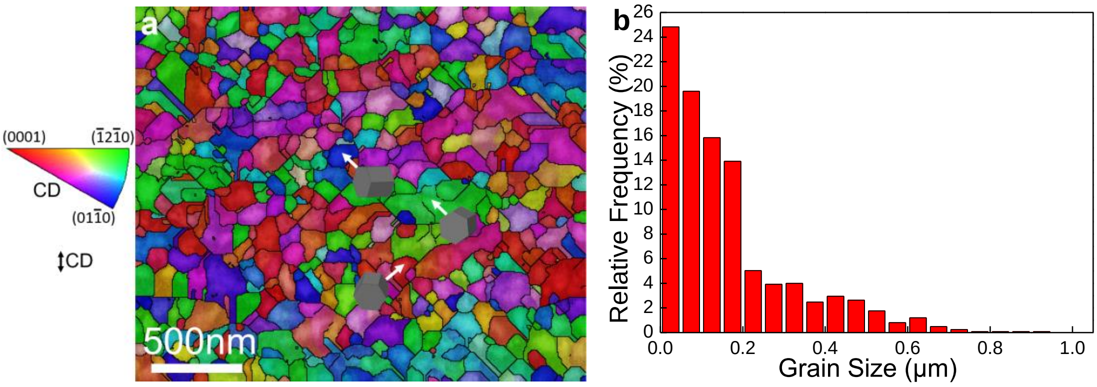

Supplementary Fig. 12 EBSD analysis of the as-prepared Ti15Zr7Cu alloy. **a,** The IPF image showing that an ultrafine-grained structure have been fabricated following the EQD strategy. Compressive direction (CD) is denoted in the figure. **b,** Grain size distribution map. Over 44% of grains are with sizes below 100 nm

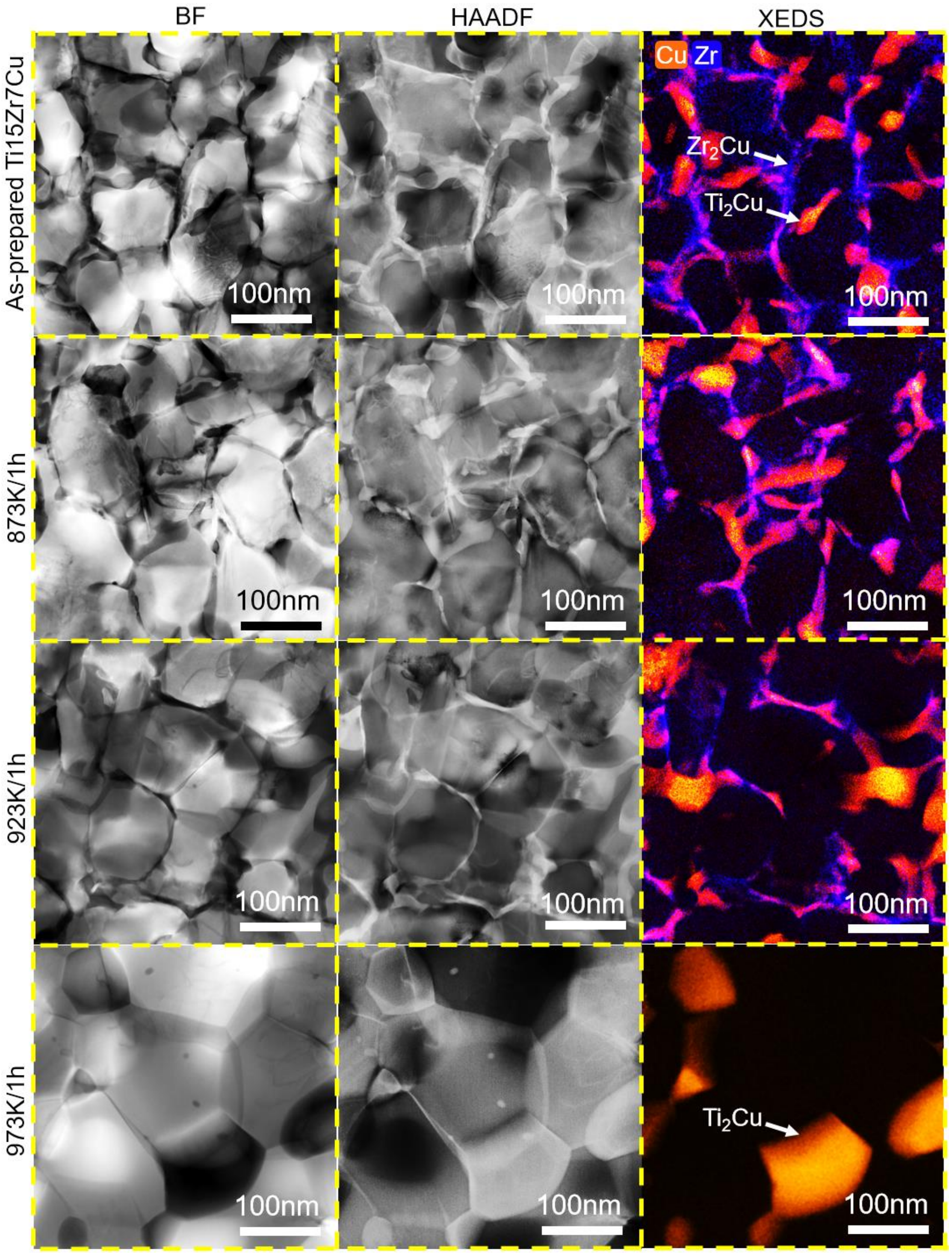

Supplementary Fig. 13 TEM observation of the Ti15Zr7Cu alloy. A DPHS nanostructure has been successfully fabricated following the QD strategy, the dual-phase shell was composed of Ti_2Cu and Zr_2Cu phase. The nanostructured alloy presents extreme high stability against annealing, the onset of grain coarsening temperature is up to 973K.

Supplementary Table1 VASP output data

Surface energy calculation results 1a

Structure model	Interface Area (Å^2)	Bulk energy (eV)	Slab energy (eV)	Surface energy (eV/Å^2)
$(\bar{1}100)_{\alpha}$	13.099	-89.772	-86.433	0.127
$(21\bar{1})_{\beta}$	13.099	-95.243	-92.309	0.112
$(0002)_{\alpha}$	42.639	-365.395	-355.044	0.121
$(0\bar{1}3)_{Ti2Cu}$	42.639	-273.133	-263.790	0.110

Interfacial energy calculation results 1b

Structure model	Interface Area (Å^2)	Model energy (eV)	Interfacial energy	
			(eV/Å^2)	(J/m^2)
$(\bar{1}100)_{\alpha}$-$(21\bar{1})_{\beta}$	13.099	-181.699	0.0137	0.219
$(0002)_{\alpha}$-$(0\bar{1}3)_{Ti2Cu}$	42.639	-628.493	0.0044	0.070

Supplementary Table2 Chemical compositions (wt.%) of alloys being investigated

	Al	V	Cu	Zr	Fe	O	C	Ti
**Ti6Al4V5Cu**	6.2	4.0	5.8	—	0.02	0.04	0.02	Bal.
**Ti6Al4V**	6.1	3.9	<0.01	—	0.07	0.09	0.03	Bal.
**Ti5Cu**	—	—	5.1	—	0.03	0.07	0.02	Bal.
**Ti15Zr7Cu**	—	—	7.0	14.8	0.03	0.02	0.01	Bal.
